# Attributing heatwave mortality to human-induced climate change in Greece: a case-crossover and attribution analysis for 2000–2019

**DOI:** 10.1186/s12940-026-01320-9

**Published:** 2026-07-11

**Authors:** Di Xi, Dimitris Evangelopoulos, Clair Barnes, Evangelos Chandakas, Constantine Vardavas, Paraskevi Katsaounou, Paolo Vineis, Filippos T Filippidis, Garyfallos Konstantinoudis

**Affiliations:** 1 MRC Centre for Environment and Health, School of Public Health, Imperial College London, London, UK; 2 Centre for Environmental Policy, Imperial College London, London, UK; 3 Department of Hygiene, Epidemiology and Medical Statistics, School of Medicine, National and Kapodistrian University of Athens, Athens, Greece; 4 First ICU Evaggelismos Hospital Athens, National and Kapodistrian University of Athens, Athens, Greece; 5 Department of Primary Care & Public Health, School of Public Health, Imperial College London, London, UK; 6 Grantham Institute for Climate Change and the Environment, Imperial College London, London, UK

**Keywords:** Extreme heat, CAR models, Climate change, Health attribution

## Abstract

**Background:**

Heatwaves increasingly threaten public health in the Mediterranean region, and Greece is among the hardest hit countries. Yet evidence on long-term adaptation, spatial vulnerability, and the contribution of human-induced climate change to heatwave-related mortality in Greece remains limited.

**Methods:**

We analysed 2,144,957 all-cause deaths in Greece (2000–2019) in people older than 65 years using a time-stratified case-crossover design. We derived population-weighted daily maximum temperatures at NUTS3 level from ERA5 reanalysis and WorldPop. We applied six heatwave definitions (HD1–HD6) varying by duration (≥ 2 or ≥ 3 days) and thresholds (90th, 95th, 99th percentiles).

We fitted Bayesian hierarchical Poisson models to estimate heatwave-mortality associations varying by space and time. We additionally adjusted for relative humidity and national holidays. We then combined these estimates with probabilistic climate-attribution methods to quantify the number and proportion of heatwave-related deaths attributable to human-induced climate change.

**Results:**

Heatwaves raised mortality consistently, with relative risks from 1.08 (95% CrI (Credible Interval): 1.07–1.09; HD1) to 1.15 (1.12–1.19; HD6). Risks increased with heatwave intensity and duration, with particularly high risks observed among females and older adults. We did not detect a consistent temporal decline in risk or marked spatial heterogeneity. Human-induced climate change accounted for 46–80% of heatwave-related deaths across definitions. The proportion attributable to climate change rose over time.

**Conclusions:**

Heatwaves already impose a major mortality burden in Greece, with more than half driven by anthropogenic climate change and little evidence of population-level adaptation. These findings call for rapid emissions reductions and targeted adaptation, including stronger heat-health warning systems and protection of vulnerable groups.

**Supplementary Information:**

The online version contains supplementary material available at https://doi.org/10.1186/s12940-026-01320-9.

## Introduction

Temperatures around the globe are increasing due to anthropogenic emissions of greenhouse gases [1]. Record-breaking temperatures observed in Europe during the 2022 summer were associated with more than 60,000 heat-related deaths [2]. Nearly half of these deaths are estimated to be attributable to human-induced climate change, with Southern European countries being disproportionately affected [3]. Focusing specifically on deaths attributable to climate change can strengthen climate risk communication and enhance “subjective attribution”, the public’s growing recognition that extreme events are linked to climate change, which is a key driver of support for climate mitigation policies [4]. Greece is among the countries in Europe most affected by increasing warming; however, there is still a lack of nationwide studies quantifying the heatwave-related health impacts of anthropogenic climate change.

Heat exposure can be defined as ambient exposure to higher temperatures or heatwaves. Heatwaves, defined as prolonged periods of unusually high temperatures above certain thresholds, have been reported to be associated with increased morbidity and mortality glo bally [2, 5–13]. Focusing on heatwaves rather than ambient temperature offers clearer cut-off points to activate targeted public health actions, such as health-heat early warnings [14]. The first ever health heat attribution study examined the extreme European heatwaves of summer 2003 and showed that anthropogenic climate change increased the risk of heatwave-related mortality by ∼70% in Central Paris and by ∼20% in London [15]. A global study reported that more than half of heatwave-related deaths in 2023 were attributable to human-induced climate change [16]. The first ever rapid health attribution study was focused on 12 European cities during a 10-day heat period in summer 2025 and reported that human-induced climate change has tripled the heatwave-related mortality toll [17]. However, all these studies rely on a single estimate of the effect of heat on mortality and do not account for possible changes over time due to adaptation. In addition, most have focused on specific events, and none has examined long-term trends in heatwave-related mortality attributable to climate change.

Using nationwide data in Greece during 2000–2019, we investigated the effect of heatwaves on all-cause mortality using six heatwave definitions. We examined temporal adaptation and spatial vulnerabilities across the different heatwave definitions, sex and age groups. We then simulated counterfactual temperature time series assuming no anthropogenic climate change and compared with the factual temperature time series to isolate the footprint of human-induced climate change on heatwave-related mortality in Greece. Finally, we analysed temporal trends in heatwave-related mortality driven by climate change over 2000–2019.

## Methods

### Data

We retrieved individual level all-cause mortality for individuals aged 65 and above during 2000–2019 in Greece from the Hellenic Statistical Authority [18]. Data included information about age, sex, region of residence classified according to the Nomenclature of Territorial Units for Statistics at level 3 (NUTS3) and the date of death [19]. We grouped the data by age (65–74, 75–84 and ≥ 85) and sex (males and females). The selection of the age subgroups was in line with the literature [20–22].

### Exposure and confounders

We retrieved daily maximum temperature for summer months (June-August) during 2000–2019 at approximately 10 km resolution from ERA5-land reanalysis dataset [23]. For each NUTS3 region, we calculated its maximum daily population-weighted temperatures using population data from WorldPop at 1 km geographical resolution (www.worldpop.org), see Supplementary Methods Sect. 1.We also obtained daily mean relative humidity from the ERA5-land reanalysis dataset and calculated the population-weighted estimate in similar way as for temperature [23].

### Heatwave definition

Heatwave is defined as a prolonged period of unusual high temperatures exceeding certain threshold within a given region, typically lasting for a duration of several consecutive days. The temperature thresholds in our analysis were region-specific, being estimated using the 90th, 95th and 99th percentiles of the distribution of the maximum temperature during 1990–2019 for each NUTS3 region. We use six heatwave definitions (HD1- HD6), to capture different combinations of heat intensity and duration, Table 1. Specifically, HD1 refers to periods of at least two consecutive days with maximum temperature above the 90th percentile, while HD2 refers to at least three consecutive days above the same threshold. Similarly, HD3 and HD4 correspond to at least two and three consecutive days above the 95th percentile, respectively, and HD5 and HD6 represent at least two and three consecutive days above the 99th percentile. We also considered a 3-day lag to account for prolonged effects, whereby a day was classified as a heatwave day if a heatwave had occurred within the preceding three days.

**Table 1:** Summary of the six heatwave definitions

Heatwave Definition (HD)	Duration (in days)	Threshold (Maximum temperature percentile^1^)
HD1	≥ 2	90th
HD2	≥ 3	90th
HD3	≥ 2	95th
HD4	≥ 3	95th
HD5	≥ 2	99th
HD6	≥ 3	99th

^1^Percentiles were calculated from the distribution of daily maximum temperatures during the study period (1990–2019)

### Counterfactual temperatures

The change in heatwave temperatures in each region associated with each degree of global warming was estimated using the probabilistic attribution protocol established by World Weather Attribution [24, 25]. Briefly, a nonstationary Generalised Extreme Value distribution is fitted to the time series of maxima, in which the location parameter of the distribution is assumed to shift linearly with global mean surface temperature. The output of this model is how much warmer each year was than a preindustrial climate with no or minimal human-caused warming. We repeated this procedure for 2 observation-based and 72 regional climate model datasets and synthesised the results. These estimates were used to constructed counterfactual temperature in a world without human-induced warming, see Supplementary Methods Sect. 2.

### Study design

We used a time-stratified case-crossover design, commonly used for analysing the effect of transient exposures [26]. The temperature on the day of mortality (event day) is compared with the temperature on non-event days. In the case-crossover design, a case serves as its own control, thus, this design automatically controls for factors that do not vary or vary slowly over time, such as sex or deprivation. We selected non-event days on the same day of week and calendar month as the event day to avoid the overlap bias [27].

### Epidemiological model

We modelled the effect of heatwave by specifying Bayesian hierarchical conditional Poisson models, with a fixed effect on the event/non-event day grouping. This has been found to provide a flexible alternative to conditional logistic regression [28]. Briefly, let $\:{Y}_{ijkl}$ taking the value 1 if it is an event and 0 otherwise on the *i*-th summer day, *j*-th NUTS3 region, *k*-th year and *l*-th event/non-event grouping. We have run the models for the different age-sex groups separately; for simplicity we omit an age-sex index from the notation:

$$\:{Y}_{ijkl}\:\sim\mathrm{P}\mathrm{o}\mathrm{i}\mathrm{s}\mathrm{s}\mathrm{o}\mathrm{n}\left({m}_{ijkl}\right)$$

$$\begin{aligned} \mathrm{l}\mathrm{o}\mathrm{g}\left({m}_{ijkl}\right)&={(b}_{1}+{w}_{j}+{v}_{k})\\&*{X}_{ijk}+{b}_{2}{Z}_{ijk}+{b}_{3}{Z}_{ik}+{\phi\:}_{l} \end{aligned}$$

where $\:{b}_{1}$ is the average heatwave effect across the regions and years, $\:{w}_{j}$ is a Gaussian autoregressive prior that accounts for spatial dependence [29], allowing the average heatwave effect to take different values in the different NUTS3 regions. The random effect $\:{v}_{k}$ is a random walk of order 2 that models temporal autocorrelation, allowing the average heatwave effect to take different values across the different years. The term $\:{b}_{2}$ accounts for relative humidity, the term $\:{b}_{3}$ for national holidays and $\:{\phi\:}_{l}$ is a fixed effect per event/non-event grouping. We accounted for national holidays by including a binary variable, with 1 indicating national holiday and otherwise 0.

We report median and 95% Credible Intervals (CrI) of the posterior of the relative heatwave mortality risk by age, sex, heatwave definition, time and space.

### Death attribution

We estimated the nationwide number of deaths attributable (AN) to each heatwave definitions during the study period, based on factual and counterfactual temperatures and stratified by age and sex group. AN were estimated by combining the heatwave relative risks with the number of heatwave days under factual and counterfactual temperature scenarios, enabling quantification of mortality attributable to anthropogenic climate change, Supplementary Methods Sect. 3. All analyses were conducted using the Integrated Nested Laplace Approximation (INLA) [30, 31].

### Sensitivity analysis

We performed a series of sensitivity analyses to test the robustness of our results. First, we allowed the heatwave threshold to vary by year and region, defining it using the 90th, 95th and 99th percentiles of the year- and region-specific maximum temperature distribution, thereby accounting for unusually hot years. Second, we modelled lagged effects jointly by including lag0 to lag3, relaxing the assumption that heatwave effects are constant across lags. Third, we tested the sensitivity of the temporal effects to the prior specification. We considered both a first-order random walk and a zero-mean normal prior with unstructured covariance, using a PC prior for the hyperparameter such that $\:P(\sigma\:>1)=0.01$. This implies that years with risks around threefold higher are unlikely and relaxes the smoothness assumption imposed by the second-order random walk.

## Results

We observed a total of 2,144,957 deaths in Greece during 2000–2019, of which 49.1% (1,052,792) were among females (Table 2). The number of deaths observed during heatwaves declined as the heatwave definitions became more extreme (Table 2). For example, there were 302,728 deaths under the least extreme definition (HD1), compared to 18,041 deaths under the most extreme definition (HD6). Descriptive statistics of the exposure are included in the Supplementary Table 1, Supplemental Figs. 1 and 2. Overall, we did not observe any consistent spatial or temporal patterns in the heatwave exposure, reflecting the choice of the percentiles in the heatwave definition.

**Table 2:** Number of all-cause deaths across age and sex group and the six different heatwave definitions in Greece from 2000 to 2019

Sex	Age	*N*	HD1	HD2	HD3	HD4	HD5	HD6
Total	Total	2,144,957	302,728	230,915	169,663	112,854	34,366	18,041
Female	Total	1,052,792	151,959	116,250	85,895	57,307	17,609	9,245
	65–74	365,681	52,794	40,639	30,064	20,083	6,215	3,246
	75–84	227,018	31,500	23,766	17,540	11,465	3,482	1,766
	≥ 85	460,093	67,665	51,845	38,291	25,759	7,912	4,233
Male	Total	1,092,165	150,769	114,665	83,768	55,547	16,757	8,796
	65–74	376,120	51,973	39,705	28,991	19,336	5,826	3,072
	75–84	420,832	57,145	43,329	31,643	20,892	6,185	3,176
	≥ 85	295,213	41,651	31,631	23,134	15,319	4,746	2,548

**Fig. 1:**
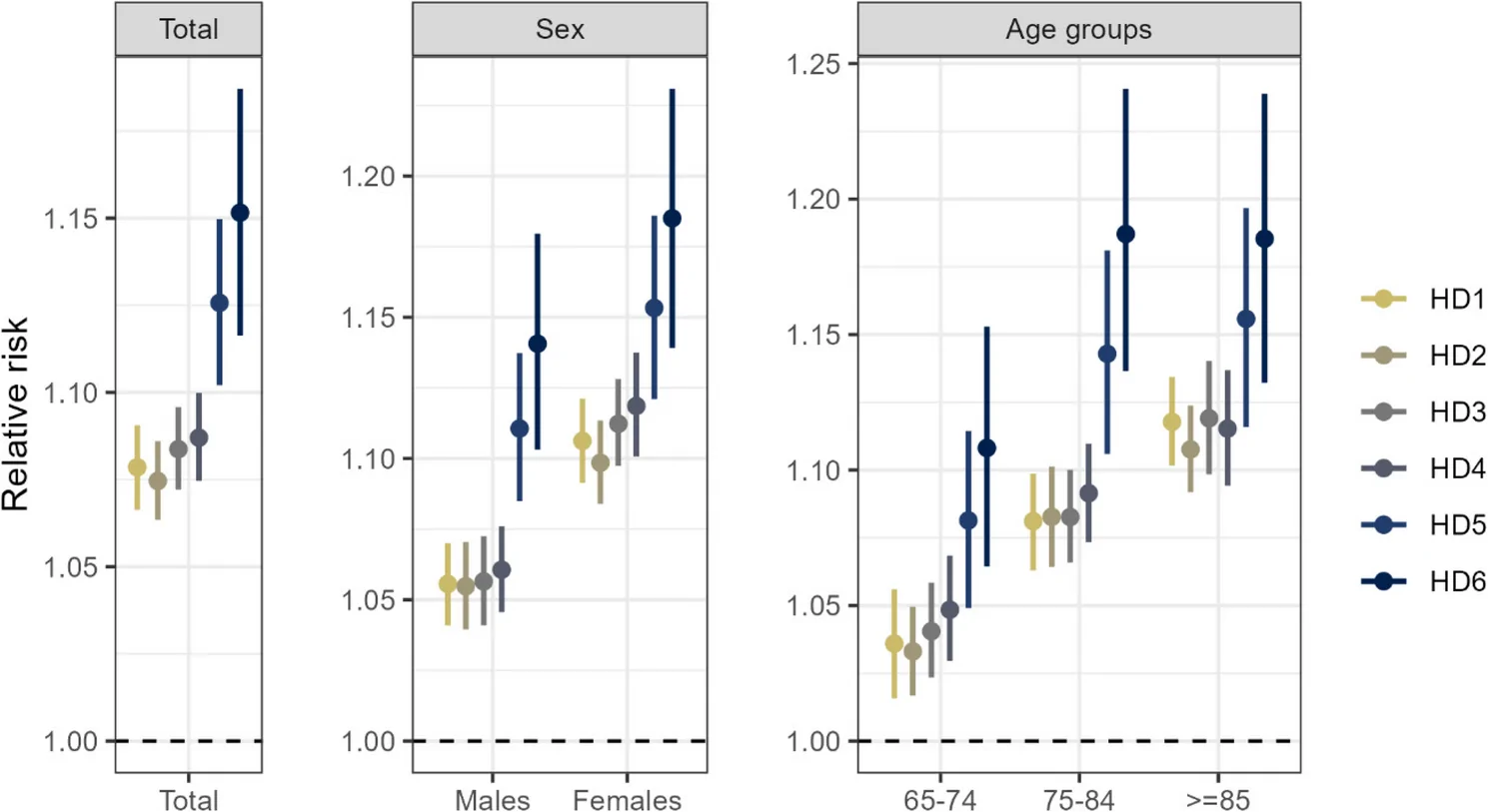
Median and 95% credible intervals of the posterior distribution of the relative heatwave risk from the fully adjusted model across the 6 heatwave definitions

Across the study period and geographical regions, we observed a strong association between heatwaves and all-cause mortality. The relative risk (RR) for mortality on heatwave days versus non-heatwave days ranged from 1.12 (95% CrI: 1.11–1.12, HD1) to 1.23 (95% CrI: 1.21–1.26, HD6) in unadjusted models (not accounting for relative humidity and national holidays), and 1.08 (95% CrI: 1.07–1.09, HD1) to 1.15 (95% CrI: 1.12–1.19, HD6) in fully adjusted models (Fig. 1, Supplementary Table 2). The effect of heatwaves on mortality increased with increasing intensity and duration (Fig. 1). However, for more extreme heatwave definitions, the number of heatwave events is lower, leading to greater uncertainty in the estimated effects (Fig. 1, Supplementary Table 2). Across the different sex and age groups, overall females and older populations were more vulnerable, with the highest RR among females 75–84 years old and those aged over 85 years: 1.21 (95% CrI: 1.15–1.29) and 1.21 (95% CrI: 1.14–1.27), respectively. (Supplementary Table 2).

**Fig. 2:**
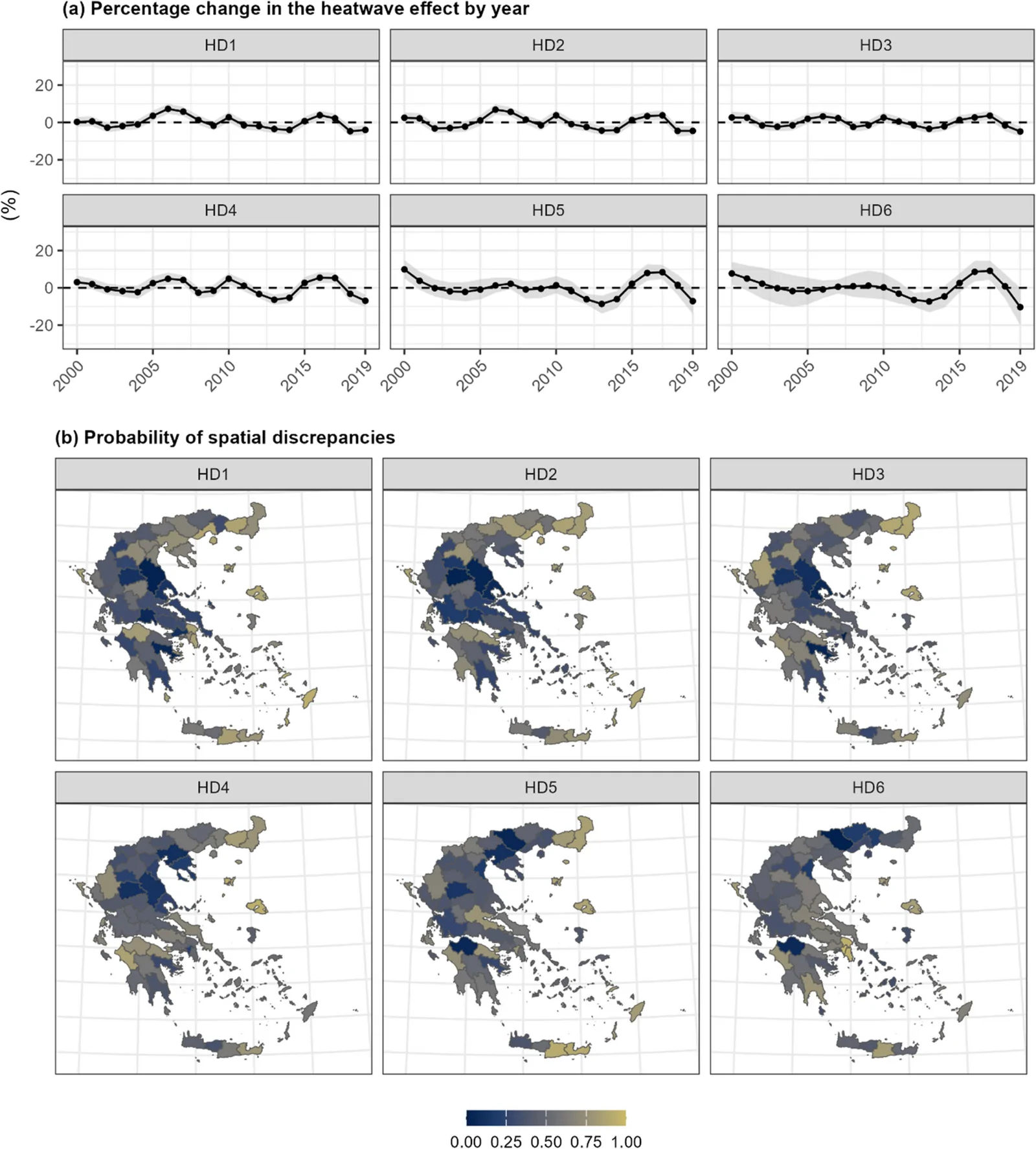
Spatiotemporal variation of the heatwave effects across the 6 heatwave definitions. Panel **(a)** shows the median percentage change in the heatwave effect across the years, whereas panel **(b)** the posterior probability that the area-specific heatwave effect is larger than the overall nationwide effect across the different heatwave definitions. Both effects were based on the fully adjusted models

Figure 2 shows the spatiotemporal trends of the heatwave effect across the six considered definitions. Panel A of Fig. 2 presents the percentage change in the heatwave effect over time. Overall, there are no consistent temporal trends in the heatwave effects across the different definitions. The smallest heatwave effect was observed in 2019, and this finding was consistent across all heatwave definitions (Panel A, Fig. 2). Panel B of Fig. 2 displays the posterior probability that the area-specific heatwave effect is larger than the overall nationwide effect across the different heatwave definitions. We found no strong evidence (i.e., posterior probability always smaller than 0.95) of spatial heterogeneity in the heatwave effect. The highest posterior probability of a spatial deviation from the overall effect was observed only for HD6 in Athinon (posterior probability = 0.98; Panel B, Fig. 2). No other regions exceeded the 0.95 threshold under any heatwave definition, suggesting that substantial spatial variation was not consistently observed across Greece.

**Fig. 3:**
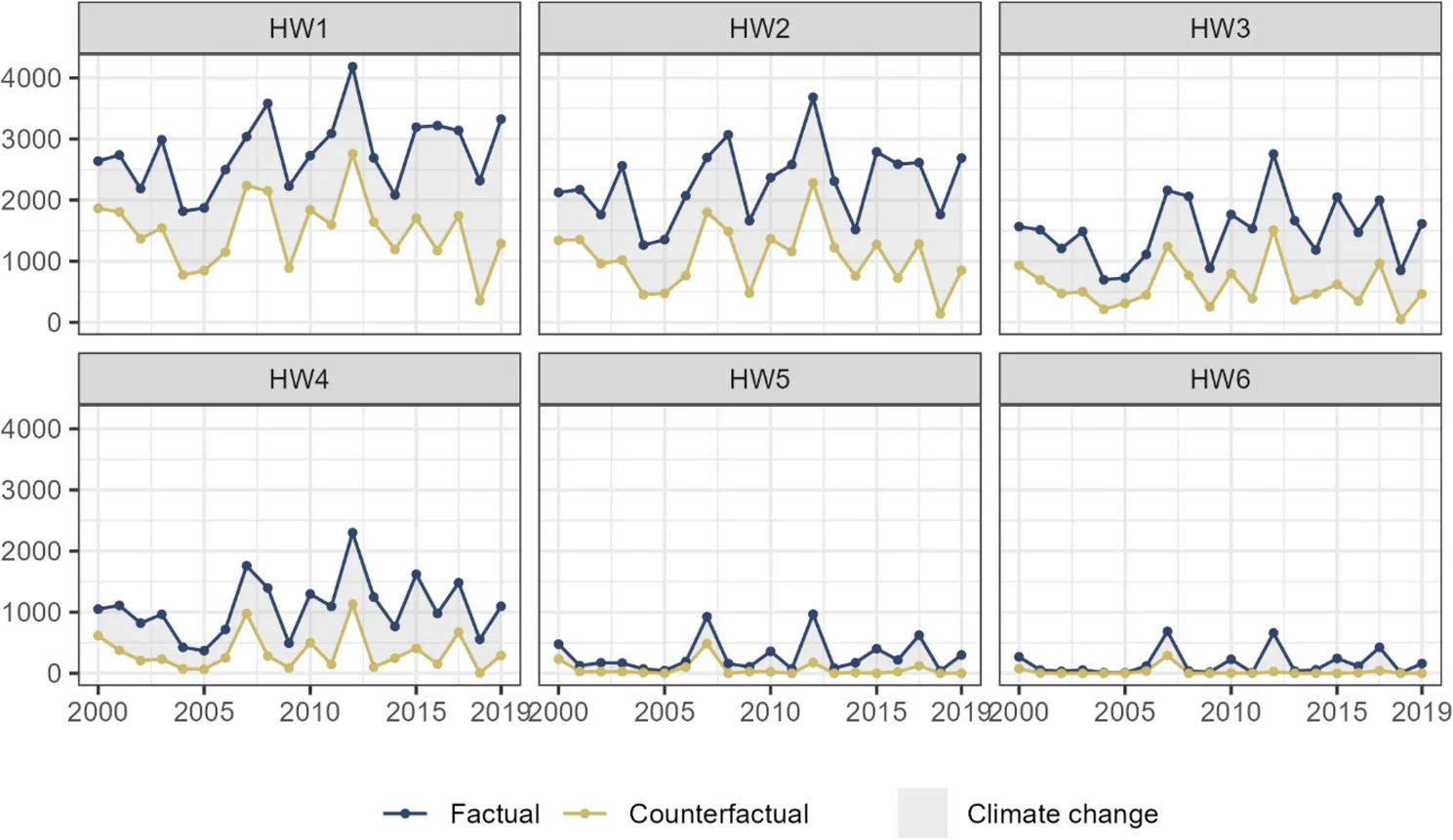
Numbers of heatwave days (y-axis) in factual and counterfactual scenarios over the study period across (x-axis) the 6 heatwave definitions

**Fig. 4:**
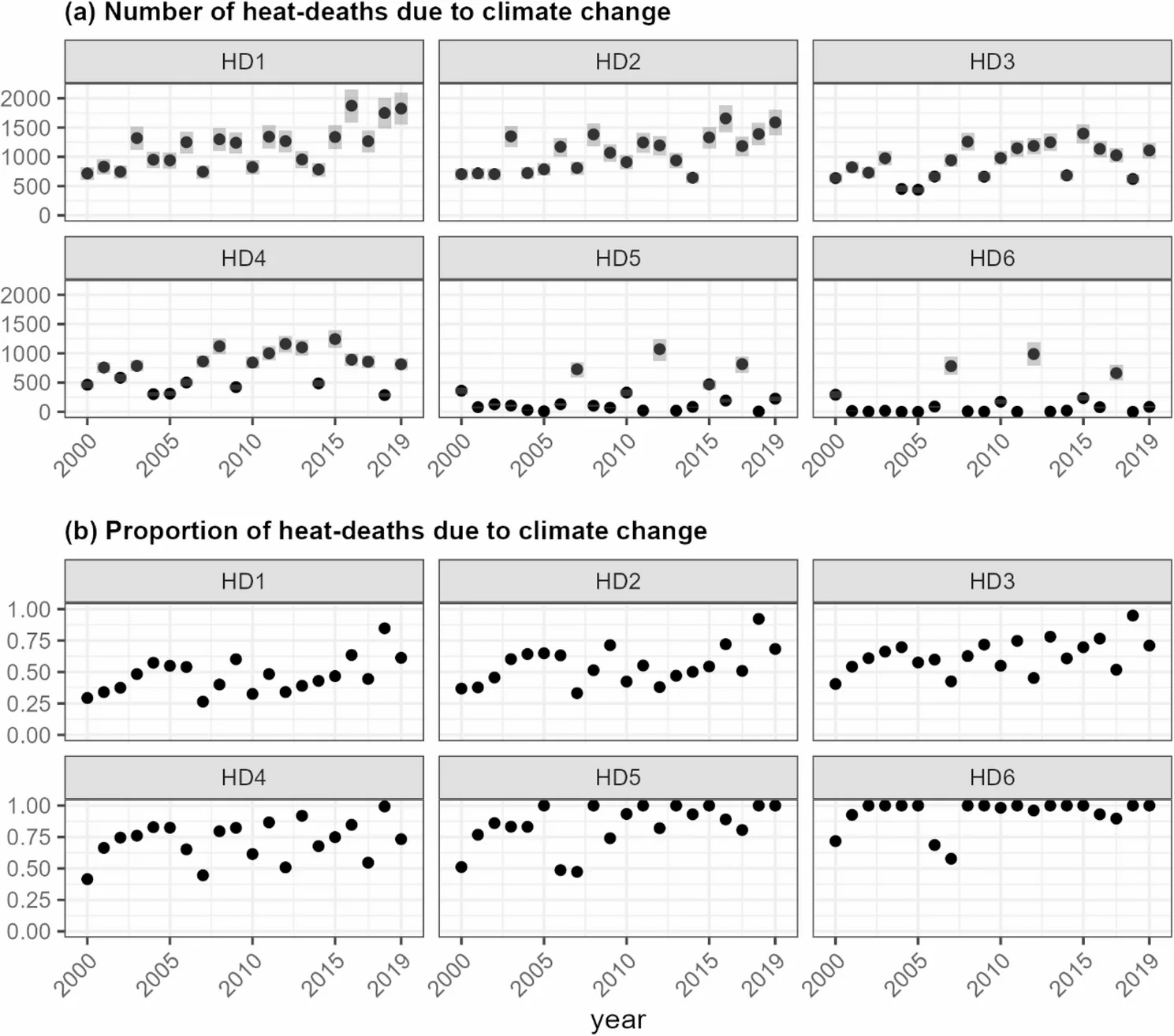
Median and 95% credible intervals of the number of heatwave-deaths panel (**a**) and relative difference of deaths panel (**b**) due to human-induced climate change across the study period

Figure 3 shows the number of heatwave days in the factual and counterfactual scenarios across the different heatwave definitions. Overall, the number of heatwave days has increased over time across all heatwave definitions. Table 3 presents the number of additional heatwave deaths and the proportion of deaths attributed to human-induced climate change. For the milder heatwave definitions, approximately half of the heatwave-related deaths during the study period were attributable to human-induced climate change. For example, under HD1, there were 50,643 (95% CrI: 43,820 − 57,662) expected heatwave-related deaths, of which 46% were attributable to climate change. In contrast, for the stricter definitions, nearly all heatwave-related deaths could be linked to anthropogenic climate change. For instance, under HD6, there were 4,325 (95% CrI: 3,425–5,290) heatwave-related deaths, 80% of which were attributable to climate change. Last, we calculated the annual number of expected heatwave-attributable deaths and the corresponding proportions attributable to climate change by year. The number of deaths showed no consistent temporal pattern, whereas the proportion increased over time (Fig. 4).

**Table 3:** Median and 95% credible intervals of number of deaths attributable to heatwaves in the factual scenario, number of heatwave deaths and the proportion of deaths attributed to human-induced climate change. The results are calculated using relative risks from the fully adjusted models

Definition	Number of death attributable to heatwave in the factual scenario	Difference in heatwave-attributable numbers between factual and counterfactual scenario	Proportion of excess death attributable to climate change (%)
HD1	50,643 (43,820, 57,662)	23,365 (20,217, 26,604)	46%
HD2	39,791 (34,138, 45,688)	21,307 (18,280, 24,464)	54%
HD3	30,040 (26,318, 33,529)	18,277 (16,012, 20,400)	61%
HD4	21,850 (19,045, 24,545)	14,798 (12,899, 16,623)	68%
HD5	6,832 (5,609, 8,001)	4,966 (4,077, 5,816)	73%
HD6	4,325 (3,425, 5,290)	3,469 (2,747, 4,243)	80%

### Sensitivity analysis

Results were consistent under the region- and year-specific threshold definition (Supplementary Figures S3-6). Models that explicitly accounted for lagged effects yielded similar findings (Supplementary Figures S7-8), although delayed heatwave effects beyond lag 3 cannot be entirely excluded. The temporal pattern of heatwave effects was also robust to alternative prior specifications (Supplementary Figures S9).

## Discussion

In this first nationwide spatiotemporal study in Greece during 2000–2019, we investigated the effect of heatwaves on all-cause mortality using different heatwave definitions. We examined temporal adaptation and spatial vulnerabilities, as well as the effect modification by age and sex. We found that heatwaves were associated with increased all-cause mortality, with higher-intensity heatwaves associated with greater risk. Older individuals and females were more vulnerable. We found weak evidence of spatiotemporal variation of the heatwave effect across the different heatwave definitions. We observed that human-induced climate change has increased the number of days exceeding the high temperature percentiles of the climatology and the associated number of heatwave-related deaths. Around half of heatwave-related deaths were attributable to human-induced climate change for less stringent heatwaves, while nearly all deaths could be linked to climate change under the stricter definitions.

The results of this study identified population groups that were more impacted than others. This demographic pattern aligns with previous heatwave-mortality studies, which consistently report elevated risks among older populations [2, 5, 7, 8, 12, 21] and females [2, 5, 8, 12, 21, 32] across different climatic and geographic contexts. Several biological and physiological mechanisms may explain this heightened susceptibility [33, 34]. Older adults often have reduced thermoregulatory capacity, diminished cardiovascular reserve, and a higher prevalence of chronic conditions that impair the ability of heat dissipation. Females may experience greater vulnerability due to differences in body composition, hormonal regulation, and higher rates of heat-sensitive comorbidities [35, 36].

In our analysis, we did not detect substantial spatial variation in heatwave-related mortality risk across regions. Previous studies focusing on mortality or hospital admissions and ambient temperature have reported some evidence of spatial effect modification that can be partly explained by environmental covariates such as green space and urbanicity [13, 37–40]. The lack of spatial heterogeneity in our study could reflect relatively homogeneous climatic conditions across much of Greece during extreme heat events, nationwide exposure patterns, or similarities in demographic, behavioural (e.g. smoking and physical activity) and health system characteristics that influence vulnerability. It may also be partly due to limitations in statistical power to detect small-scale geographic differences.

Our results provide weak evidence of population-wide adaptation to heatwaves in Greece over the study period. This limited adaptation contrasts with findings from other Mediterranean European countries, such as Spain [41–43], where previous studies have reported a reduction in heatwave-related mortality risk and attributable deaths over time. A multi-country study also found a decrease in heatwave-related mortality impacts over the past decades, beyond what would be expected from adaptation alone given the observed warming in most of the studied countries [44]. However, there was only weak evidence of adaptation in Australia and Brazil [44]. A study using partly overlapping data focusing on Thessaloniki (1999–2018) ,Greece’s second-largest city, and Athens (1999–2019), the capital, reported steeper temperature-mortality curves in Athens during 2011–2019 compared to 1999–2007, while curves in Thessaloniki remained largely unchanged [45].

This discrepancy may reflect methodological differences from previous studies, including the choice of exposure metric (heatwaves versus ambient temperature), the selection of percentiles and duration criteria, the consideration of lag periods, as well as differences in study period, study region, and statistical model. The consistency of our results with the previous Greek study further supports our conclusion of limited adaptation to heat in Greece. In contrast, the evidence of adaptation reported in other Mediterranean countries, such as Italy and Spain [44, 46], may reflect the greater effectiveness of their heat-health warning systems, better hospital preparedness, and more comprehensive public health interventions. In Greece, the apparent lack of adaptation could be linked to less efficient heat-health alert systems, limited investment in green space and urban cooling strategies, and insufficient integration of air pollution reduction policies with heat mitigation plans. Strengthening these areas could enhance resilience to rising temperatures and reduce the future health burden of heat.

Our study is consistent with previous studies quantifying the impact of human-induced climate change on heatwave-related mortality, especially under milder heatwave definitions. For example, under HD1 and HD2, 46% and 54% of heatwave-related death can be attributable to climate change. This is in line with a global study reporting that 54.3% of heatwave-related deaths were attributable to human-induced climate change [16], as well as a European study in the summer of 2022, which found that 56% of observed heat-related deaths could be attributed to climate change [2]. Similarly, a study in Switzerland estimated this proportion at 60% [47]. Furthermore, our study reported most heatwave-related deaths can be linked to climate change under the stricter definitions. For instance, under HD4, 68% of heatwave-related death can be attributable to climate change, and under HD6, this proportion reached 80%. This number is consistent with a recent rapid health attribution study focusing on 854 cities in Europe, reporting 70% of the expected heatwave-related mortality to be attributed to climate change [48]. The high attributable proportions observed for the most extreme heatwave definitions should be interpreted with caution. These estimates largely reflect that such rare events are much less frequent in the cooler counterfactual climate. Thus, this metric primarily captures the sensitivity of extreme heatwave occurrence to anthropogenic warming rather than differences in the underlying heatwave-mortality relative risk.

The main strength of this study is the availability of a large nationwide dataset with granular spatial information in Greece during 2000–2019 making the results generalisable to both urban and rural settings. The availability of individual data minimises ecological bias [49]. We proposed a Bayesian spatiotemporal framework to estimate the effect of heatwaves in space and time by borrowing information from spatial and temporal neighbours. This aims to smooth the heatwave effects towards local and global means and accounts for data sparsity. Nevertheless, as the heatwaves are relatively rare events, this study had limited power to detect changes in space for more extreme heatwave definitions.

Our study has some limitations. Residential temperature does not reflect the actual temperature exposure of an individual, as individuals are exposed to different temperatures during the day. In addition, the outdoor temperature, does not always reflect the actual temperature exposure in indoor spaces. Nevertheless, in line with most of the studies in this field and given the lack of more precise individual exposure data, we used residential temperature outdoors as a proxy for the individual exposure. Our analysis lacked information on additional individual-level factors that could influence vulnerability, including access to air conditioning, smoking status and medication use. Due to the lack of daily air pollution data at high geographical resolution, we were unable to adjust for air pollution in our analyses, which represents an important limitation. Previous literature suggests that some pollutants, particularly ozone, may partly mediate the effect of heat on mortality, as high temperatures can enhance ozone formation during heatwaves [50]. Consequently, adjustment for such pollutants could attenuate the estimated heatwave effect by accounting for pathways operating through air pollution [51]. In contrast, the role of other pollutants, such as NO_2_ and PM_2.5_, may be more complex [9]. For instance, if PM_2.5_ acts as a confounder, failure to adjust for it could result in an overestimation of the heatwave-mortality association. A previous study reported that adjustment for PM_10_ attenuated heatwave effect estimates by around 30% [9]. Furthermore, air pollution may exacerbate the heatwave effect [52], but assessment of this potential interaction was beyond the scope of the present study. Additionally, in our time-stratified case-crossover designs, some control days may occur after the death event. However, this sampling is not expected to introduce bias because environmental exposures are exogenous to individual mortality. Last, our definition of prolonged heatwaves to account for lags, assumes a common effect across lag0 and lag3 and therefore does not identify the lag structure explicitly; however, analyses based on jointly modelled lags produced comparable cumulative effects.

## Conclusion

Our findings highlight the substantial health burden attributable to heatwaves in Greece, particularly among older adults and females. As climate change increases the frequency and intensity of extreme heat events, proactive adaptation measures, such as heat–health warning systems, community outreach, and tailored guidance for vulnerable populations, will be critical to reducing mortality. The absence of a temporal decline in heatwave-related mortality risk suggests limited adaptation or ineffective implementation of existing measures. Our counterfactual analysis indicates that a significant proportion of heatwave-related deaths are attributable to human-induced climate change, highlighting the importance of both mitigation policies to curb greenhouse gas emissions and adaptation strategies to protect public health. Protecting vulnerable populations through early warning systems, public health preparedness, and community-based interventions, together with mitigation actions such as decarbonizing the energy sector and expanding urban green spaces, will be essential to reduce future heatwave-related deaths in Greece.

## Supplementary Information

Supplementary Material 1.

## Data Availability

No datasets were generated or analysed during the current study.

## Declarations

### Data sharing

The data used in this study were obtained from the Hellenic Statistical Authority (ELSTAT). Access to these data is subject to restrictions and was granted for the purposes of this research under specific authorization. The data are not publicly available but can be requested directly from ELSTAT, subject to their data access policies.

Air temperature at 2 m for was retrieved from ERA5-land https://cds.climate.copernicus.eu/cdsapp#!/dataset/reanalysis-era5-land?tab=form.

## References

[1] Rocha J, Oliveira S, Viana CM, Ribeiro AI. Climate change and its impacts on health, environment and economy. In: One Health. Elsevier; 2022:253–79. Available from: https://linkinghub.elsevier.com/retrieve/pii/B9780128227947000095. . Cited 23 Apr 2025.

[2] Ballester J, Quijal-Zamorano M, Méndez Turrubiates RF, Pegenaute F, Herrmann FR, Robine JM, et al. Heat-related mortality in Europe during the summer of 2022. Nat Med. 2023;29(7):1857–66. 10.1038/s41591-023-02419-z. PMC10353926 37429922

[3] Vicedo-Cabrera AM, Scovronick N, Sera F, Royé D, Schneider R, Tobias A, et al. The burden of heat-related mortality attributable to recent human-induced climate change. Nat Clim Chang. 2021;11(6):492–500. 10.1038/s41558-021-01058-x. PMC7611104 34221128

[4] Cologna V, Meiler S, Kropf CM, Lüthi S, Mede NG, Bresch DN, et al. Extreme weather event attribution predicts climate policy support across the world. Nat Clim Chang. 2025;15(7):725–35. 10.1038/s41558-025-02372-4. PMC12237696 40641510

[5] Yang J, Yin P, Sun J, Wang B, Zhou M, Li M, et al. Heatwave and mortality in 31 major Chinese cities: Definition, vulnerability and implications. Sci Total Environ. 2019;649:695–702. 10.1016/j.scitotenv.2018.08.332. 30176480

[6] Xu Z, FitzGerald G, Guo Y, Jalaludin B, Tong S. Impact of heatwave on mortality under different heatwave definitions: A systematic review and meta-analysis. Environ Int. 2016;89–90:193–203. 10.1016/j.envint.2016.02.007. 26878285

[7] Kim Yook, Lee W, Kim H, Cho Y. Social isolation and vulnerability to heatwave-related mortality in the urban elderly population: A time-series multi-community study in Korea. Environ Int. 2020;142:105868. 10.1016/j.envint.2020.105868. 32593050

[8] Tong S, FitzGerald G, Wang XY, Aitken P, Tippett V, Chen D, et al. Exploration of the health risk-based definition for heatwave: A multi-city study. Environ Res. 2015;142:696–702. 10.1016/j.envres.2015.09.009. 26386208

[9] Analitis A, Michelozzi P, D’Ippoliti D, de’Donato F, Menne B, Matthies F, et al. Effects of Heat Waves on Mortality: Effect Modification and Confounding by Air Pollutants. Epidemiology. 2014;25(1):15–22. 10.1097/EDE.0b013e31828ac01b. 24162013

[10] Anderson BG, Bell ML, Weather-Related, Mortality. How Heat, Cold, and Heat Waves Affect Mortality in the United States. Epidemiology. 2009;20(2):205–13. 10.1097/EDE.0b013e318190ee08. PMC3366558 19194300

[11] Chen K, Bi J, Chen J, Chen X, Huang L, Zhou L. Influence of heat wave definitions to the added effect of heat waves on daily mortality in Nanjing, China. Sci Total Environ. 2015;506–507:18–25. 10.1016/j.scitotenv.2014.10.092. 25460935

[12] Ma W, Zeng W, Zhou M, Wang L, Rutherford S, Lin H, et al. The short-term effect of heat waves on mortality and its modifiers in China: An analysis from 66 communities. Environ Int. 2015;75:103–9. 10.1016/j.envint.2014.11.004. 25461419

[13] Xing Y, Xu R, Xu Z, Li Z, Yang Z, Zhang Y, et al. Mapping heat-related morbidity burden attributable to human-induced climate change across 460 communities of Victoria, Australia. Environ Health. 2026. 10.1186/s12940-026-01289-5. PMC13126718 41862912

[14] McGregor GR, Bessemoulin P, Ebi KL, Menne B, editors. Heatwaves and health: guidance on warning-system development. Geneva: World Meteorological Organization: World Health Organization;; 2015.

[15] Mitchell D, Heaviside C, Vardoulakis S, Huntingford C, Masato G, Guillod P. Attributing human mortality during extreme heat waves to anthropogenic climate change. Environ Res Lett. 2016;11(7):074006. 10.1088/1748-9326/11/7/074006.

[16] Hundessa S, Huang W, Xu R, Yang Z, Zhao Q, Gasparrini A, et al. Global excess deaths associated with heatwaves in 2023 and the contribution of human-induced climate change. Innov. 2025;6(10):101110. 10.1016/j.xinn.2025.101110. PMC7618246 41084606

[17] Climate-change-tripled-heat-related-deaths-in-early-summer-European-heatwave.pdf. Available from: https://www.imperial.ac.uk/media/imperial-college/grantham-institute/public/publications/institute-reports-and-analytical-notes/Climate-change-tripled-heat-related-deaths-in-early-summer-European-heatwave.pdf. Cited 2 Jan 2026.

[18] Main Page ELSTAT - ELSTAT. Available from: https://www.statistics.gr/en/home%20/. Cited 6 Nov 2025.

[19] Regulation (EC). No 1059/2003 of the European Parliament and of the Council of 26 May 2003 on the establishment of a common classification of territorial units for statistics (NUTS). 2019. Available from: http://data.europa.eu/eli/reg/2003/1059/2019-11-13.

[20] Bunker A, Wildenhain J, Vandenbergh A, Henschke N, Rocklöv J, Hajat S, et al. Effects of Air Temperature on Climate-Sensitive Mortality and Morbidity Outcomes in the Elderly; a Systematic Review and Meta-analysis of Epidemiological Evidence. EBioMedicine. 2016;6:258–68. 10.1016/j.ebiom.2016.02.034. PMC4856745 27211569

[21] Cleland SE, Steinhardt W, Neas LM, Jason West J, Rappold AG. Urban heat island impacts on heat-related cardiovascular morbidity: A time series analysis of older adults in US metropolitan areas. Environ Int. 2023;178:108005. 10.1016/j.envint.2023.108005. PMC10599453 37437316

[22] Gasparrini A, Armstrong B, Kovats S, Wilkinson P. The effect of high temperatures on cause-specific mortality in England and Wales. Occup Environ Med. 2012;69(1):56–61. 10.1136/oem.2010.059782. 21389012

[23] Copernicus Climate Change Service. ERA5-Land hourly data from 2001 to present. ECMWF. 2019. Available from: https://cds.climate.copernicus.eu/doi/10.24381/cds.e2161bac. . Cited 25 Sep 2023.

[24] Van Oldenborgh GJ, Van Der Wiel K, Kew S, Philip S, Otto F, Vautard R, et al. Pathways and pitfalls in extreme event attribution. Clim Change. 2021;166(1–2):13. 10.1007/s10584-021-03071-7.

[25] Philip S, Kew S, Van Oldenborgh GJ, Otto F, Vautard R, Van Der Wiel K, et al. A protocol for probabilistic extreme event attribution analyses. Adv Stat Clim Meteorol Oceanogr. 2020;6(2):177–203. 10.5194/ascmo-6-177-2020.

[26] Maclure M. The Case-Crossover Design: A Method for Studying Transient Effects on the Risk of Acute Events. Am J Epidemiol. 1991;133(2):144–53. 10.1093/oxfordjournals.aje.a115853. 1985444

[27] Janes H, Sheppard L, Lumley T. Overlap bias in the case-crossover design, with application to air pollution exposures. Statist Med. 2005;24(2):285–300. 10.1002/sim.1889. 15546133

[28] Armstrong BG, Gasparrini A, Tobias A. Conditional Poisson models: a flexible alternative to conditional logistic case cross-over analysis. BMC Med Res Methodol. 2014;14(1):122. 10.1186/1471-2288-14-122. PMC4280686 25417555

[29] Riebler A, Sørbye SH, Simpson D, Rue H. An intuitive Bayesian spatial model for disease mapping that accounts for scaling. Stat Methods Med Res. 2016;25(4):1145–65. 10.1177/0962280216660421. 27566770

[30] Rue H, Martino S, Chopin N. Approximate Bayesian Inference for Latent Gaussian models by using Integrated Nested Laplace Approximations. J Royal Stat Soc Ser B: Stat Methodol. 2009;71(2):319–92. 10.1111/j.1467-9868.2008.00700.x.

[31] Rue H, Riebler A, Sørbye SH, Illian JB, Simpson DP, Lindgren FK. Bayesian Computing with INLA: A Review. Annu Rev Stat Appl. 2017;4(1):395–421. 10.1146/annurev-statistics-060116-054045.

[32] Basagaña X, Sartini C, Barrera-Gómez J, Dadvand P, Cunillera J, Ostro B, et al. Heat Waves and Cause-specific Mortality at all Ages. Epidemiology. 2011;22(6):765–72. 10.1097/EDE.0b013e31823031c5. 21968768

[33] Balmain BN, Sabapathy S, Louis M, Morris NR. Aging and Thermoregulatory Control: The Clinical Implications of Exercising under Heat Stress in Older Individuals. Biomed Res Int. 2018;2018:1–12. 10.1155/2018/8306154. PMC6098859 30155483

[34] McKenna ZJ, Foster J, Atkins WC, Belval LN, Watso JC, Jarrard CP, et al. Age alters the thermoregulatory responses to extreme heat exposure with accompanying activities of daily living. J Appl Physiol. 2023;135(2):445–55. 10.1152/japplphysiol.00285.2023. PMC10538984 37410904

[35] Zhou Y, Larochelle L, Khan FA, Pilote L. Sex differences in the impact of extreme heat on cardiovascular disease outcomes: a systematic review and meta-analysis. Environ Health. 2025;24(1):20. 10.1186/s12940-025-01175-6. PMC11992858 40221760

[36] Kaciuba-Uscilko H, Grucza R Gender differences in thermoregulation. Curr Opin Clin Nutr Metab Care. 2001;4(6):533–6. 10.1097/00075197-200111000-00012. 11706289

[37] Konstantinoudis G, Minelli C, Vicedo Cabrera AM, Ballester J, Gasparrini A, Blangiardo M. Ambient heat exposure and COPD hospitalisations in England: A nationwide case-crossover study during 2007–2018 [preprint]. Public and Global Health; 2021. Available from: http://medrxiv.org/lookup/doi/10.1101/2021.10.19.21265213. Cited Oct 24 2022. 10.1136/thoraxjnl-2021-218374 PMC9606528 35459745

[38] Konstantinoudis G, Minelli C, Lam HCY, Fuertes E, Ballester J, Davies B, et al. Asthma hospitalisations and heat exposure in England: a case–crossover study during 2002–2019. Thorax. 2023;78(9):875–81. 10.1136/thorax-2022-219901. PMC10447396 37068951

[39] Bennett JE, Blangiardo M, Fecht D, Elliott P, Ezzati M. Vulnerability to the mortality effects of warm temperature in the districts of England and Wales. Nat Clim Change. 2014;4(4):269–73. 10.1038/nclimate2123.

[40] Chen X, Blangiardo M, Gascoigne C, Konstantinoudis G. Modelling the Spatially Varying Non-Linear Effects of Heat Exposure. arXiv. 2025. Available from: https://arxiv.org/abs/2502.20745. Cited Nov 7 2025.

[41] Achebak H, Devolder D, Ballester J. Trends in temperature-related age-specific and sex-specific mortality from cardiovascular diseases in Spain: a national time-series analysis. Lancet Planet Health. 2019;3(7):e297–306. 10.1016/S2542-5196(19)30090-7. 31230996

[42] Ordanovich D, Tobías A, Ramiro D. Temporal variation of the temperature-mortality association in Spain: a nationwide analysis. Environ Health. 2023;22(1):5. 10.1186/s12940-022-00957-6. PMC9838025 36635705

[43] Todd N, Valleron AJ. Space–Time Covariation of Mortality with Temperature: A Systematic Study of Deaths in France, 1968–2009. Environ Health Perspect. 2015;123(7):659–64. 10.1289/ehp.1307771. PMC4492259 25803836

[44] Vicedo-Cabrera AM, Sera F, Guo Y, Chung Y, Arbuthnott K, Tong S, et al. A multi-country analysis on potential adaptive mechanisms to cold and heat in a changing climate. Environ Int. 2018;111:239–46. 10.1016/j.envint.2017.11.006. 29272855

[45] Psistaki K, Kouis P, Michanikou A, Yiallouros PK, Papatheodorou SI, Paschalidou AΚ. Temporal trends in temperature-related mortality and evidence for maladaptation to heat and cold in the Eastern Mediterranean region. Sci Total Environ. 2024;943:173899. 10.1016/j.scitotenv.2024.173899. 38862043

[46] de’Donato F, Scortichini M, De Sario M, De Martino A, Michelozzi P. Temporal variation in the effect of heat and the role of the Italian heat prevention plan. Public Health. 2018;161:154–62. 10.1016/j.puhe.2018.03.030. 29751981

[47] Vicedo-Cabrera AM, De Schrijver E, Schumacher DL, Ragettli MS, Fischer EM, Seneviratne SI. The footprint of human-induced climate change on heat-related deaths in the summer of 2022 in Switzerland. Environ Res Lett. 2023;18(7):074037. 10.1088/1748-9326/ace0d0. PMC7615730 38476980

[48] Barnes C, Konstantinoudis G, Masselot P, Mistry M, Gasparrini A, Vicedo-Cabrera AM et al. Summer heat deaths in 854 European cities more than tripled due to climate change. 2025. 10.25560/123873.

[49] Greenland S. Ecologic versus individual-level sources of bias in ecologic estimates of contextual health effects. Int J Epidemiol. 2001;30(6):1343–50. 10.1093/ije/30.6.1343. 11821344

[50] Pu X, Wang TJ, Huang X, Melas D, Zanis P, Papanastasiou DK, et al. Enhanced surface ozone during the heat wave of 2013 in Yangtze River Delta region, China. Sci Total Environ. 2017;603–604:807–16. 10.1016/j.scitotenv.2017.03.056. 28442137

[51] Gao J, Wood D, Katsouyanni K, Benmarhnia T, Evangelopoulos D. The synergistic and mediating effects of ozone on associations between high temperature, heatwaves and mortality in the Greater London area between 2010 and 2018. Environ Res. 2025;277:121577. 10.1016/j.envres.2025.121577. 40209985

[52] Anenberg SC, Haines S, Wang E, Nassikas N, Kinney PL. Synergistic health effects of air pollution, temperature, and pollen exposure: a systematic review of epidemiological evidence. Environ Health. 2020;19(1):130. 10.1186/s12940-020-00681-z. PMC7720572 33287833

