# Supplementary material for: Attributing heatwave mortality to human-induced climate change in Greece: a case-crossover and attribution analysis for 2000–2019
Source: Environ Health. 2026 Jul 11;25:78. doi: 10.1186/s12940-026-01320-9 (PMC13640306; doi:10.1186/s12940-026-01320-9)
Supplement: Supplementary file 1 [file 12940_2026_1320_MOESM1_ESM.docx]

Online Supplement

**Attributing heatwave mortality to human-induced climate change in Greece: a case-crossover and attribution analysis for 2000-2019**

**Authors**: Di Xi^1^, Dimitris Evangelopoulos^1^, Clair Barnes^2^, Evangelos Chandakas^1^, Constantine Vardavas^3^, Paraskevi Katsaounou^4^, Paolo Vineis^1^, Filippos T Filippidis^5^, Garyfallos Konstantinoudis^6*^,

**Affiliations**

1 MRC Centre for Environment and Health, School of Public Health, Imperial College London

2 Centre for Environmental Policy, Imperial College London

3 Department of Hygiene, Epidemiology and Medical Statistics, School of Medicine, National and Kapodistrian University of Athens, Athens, Greece

4 First ICU Evaggelismos Hospital Athens, National and Kapodistrian University of Athens, Athens, Greece

5 Department of Primary Care & Public Health, School of Public Health, Imperial College London

6 Grantham Institute for Climate Change and the Environment, Imperial College London

* Corresponding author, email: [g.konstantinoudis@imperial.ac.uk](mailto:g.konstantinoudis@imperial.ac.uk)

Table of Contents

[Supplementary Tables	4](#_Toc232156858)

[Supplementary Table 1. Descriptive statistics of population-weighted daily maximum temperature and relative humidity	4](#_Toc232156859)

[Supplementary Table 2. Relative risk estimates for overall effect and effect modification by age and sex	7](#_Toc232156860)

[Supplementary Figures	9](#_Toc232156861)

[Supplementary Figure 1. Annual heatwave intensity and frequency under each definition.	9](#_Toc232156862)

[Supplementary Figure 2. Total number of heatwave days under each definition over the study period in each NUTS3 region.	10](#_Toc232156863)

[Supplementary Figure 3. Median and 95% credible intervals of the posterior distribution of the relative heatwave risk from the fully adjusted model across the 6 heatwave definitions, using year- and region-specific thresholds.	11](#_Toc232156864)

[Supplementary Figure 4. Spatiotemporal variation of the heatwave effects across the 6 heatwave definitions using year- and region-specific thresholds. Panel A shows the median percentage change in the heatwave effect across the years, whereas panel B the posterior probability that the area-specific heatwave effect is larger than the overall nationwide effect across the different heatwave definitions. Both effects were based on the fully adjusted models.	12](#_Toc232156865)

[Supplementary Figure 5. Numbers of heatwave days (y-axis) in factual and counterfactual scenarios over the study period across (x-axis) the 6 heatwave definitions using year- and region-specific thresholds.	14](#_Toc232156866)

[Supplementary Figure 6. Median and 95% credible intervals of the number of heatwave-deaths and relative difference of deaths due to human-induced climate change across the study period, with heatwave definitions using year- and region-specific thresholds.	15](#_Toc232156867)

[Supplementary Figure 7. Heatwave effects across lag0 to lag3 and the common-effect lag0–3 estimate for six heatwave definitions	16](#_Toc232156868)

[Supplementary Figure 8. Comparison of common-effect and jointly modelled cumulative lag specifications	17](#_Toc232156869)

[Supplementary Figure 9. Temporal heatwave effects under alternative prior specifications: first-order random walk and zero-mean normal prior with unstructured covariance	18](#_Toc232156870)

[Supplementary Texts	19](#_Toc232156871)

[Supplementary Methods Section 1: Justification of population-weighted temperature	19](#_Toc232156872)

[Supplementary Methods Section 2: Counterfactual temperatures	20](#_Toc232156873)

[Supplementary Methods Section 3: Death attribution	22](#_Toc232156874)

# Supplementary Tables

## Supplementary Table 1. Descriptive statistics of population-weighted daily maximum temperature and relative humidity

| **NUTS3 region** | **Population-weighted daily maximum temperature (lag0)** | | **mean relative humidity (%, lag0)** | |
| --- | --- | --- | --- | --- |
|  | **Mean (SD)** | **Median [Min, Max]** | **Mean (SD)** | **Median [Min, Max]** |
| N. ACHAIAS | 19.2 (8.01) | 18.2 [-2.21, 39.4] | 73.8 (12.3) | 76.2 [30.2, 96.1] |
| N. ANATOLIKIS ATTIKIS | 21.3 (8.33) | 20.6 [-1.12, 43.7] | 65.2 (14.1) | 66.3 [23.3, 93.8] |
| N. ARGOLIDAS | 21.1 (8.58) | 20.2 [-1.34, 43.0] | 66.8 (14.2) | 68.6 [24.8, 97.6] |
| N. ARKADIAS | 17.9 (8.46) | 17.1 [-4.53, 38.7] | 71.3 (15.0) | 73.5 [24.8, 98.3] |
| N. ARTAS | 18.8 (8.02) | 18.1 [-2.40, 39.9] | 76.6 (11.3) | 77.1 [32.0, 99.4] |
| N. ATHINON | 21.2 (8.37) | 20.3 [-0.967, 43.2] | 66.3 (14.0) | 67.7 [24.3, 94.9] |
| N. CHALKIDIKIS | 18.6 (7.78) | 18.2 [-3.38, 38.0] | 71.2 (10.8) | 71.3 [34.4, 95.6] |
| N. CHANION | 18.8 (6.13) | 18.2 [2.34, 35.2] | 71.4 (10.4) | 72.9 [22.8, 94.6] |
| N. CHIOU | 19.6 (7.22) | 18.9 [0.0571, 35.7] | 67.0 (11.1) | 67.1 [31.5, 93.6] |
| N. DODEKANISON | 20.6 (5.73) | 20.0 [4.19, 33.3] | 71.3 (8.90) | 72.8 [37.9, 92.1] |
| N. DRAMAS | 17.8 (8.52) | 17.5 [-4.86, 38.3] | 73.8 (11.4) | 74.4 [32.2, 99.1] |
| N. DYTIKIS ATTIKIS | 20.2 (8.47) | 19.3 [-2.10, 41.8] | 66.5 (14.3) | 67.7 [23.3, 96.8] |
| N. ETOLOAKARNANIAS | 19.6 (7.73) | 18.6 [-0.941, 39.9] | 74.3 (10.6) | 75.3 [35.1, 97.7] |
| N. EVROU | 18.9 (9.37) | 18.5 [-9.06, 40.7] | 69.0 (13.6) | 70.1 [25.7, 96.8] |
| N. EVRYTANIAS | 15.2 (8.30) | 15.0 [-9.27, 35.8] | 75.1 (14.1) | 76.3 [26.5, 99.4] |
| N. EVVIAS | 19.9 (7.76) | 19.5 [-1.14, 40.9] | 70.2 (11.8) | 70.6 [24.4, 96.4] |
| N. FLORINAS | 15.0 (9.49) | 14.9 [-14.0, 37.2] | 70.6 (15.2) | 71.4 [25.5, 99.6] |
| N. FOKIDAS | 17.5 (8.20) | 16.9 [-5.30, 37.8] | 72.5 (12.6) | 74.4 [26.6, 96.5] |
| N. FTHIOTIDAS | 19.1 (8.82) | 18.5 [-4.79, 41.0] | 70.5 (14.9) | 72.0 [23.8, 98.2] |
| N. GREVENON | 16.8 (9.23) | 16.6 [-8.83, 38.4] | 67.2 (15.7) | 67.5 [24.9, 99.0] |
| N. ILIAS | 20.6 (7.37) | 19.4 [0.810, 39.8] | 74.4 (9.80) | 75.9 [31.3, 94.9] |
| N. IMATHIAS | 18.2 (8.93) | 17.9 [-7.69, 39.6] | 70.2 (13.8) | 70.8 [30.2, 98.2] |
| N. IOANNINON | 17.1 (8.72) | 16.5 [-5.67, 39.1] | 74.4 (13.6) | 75.1 [27.9, 99.8] |
| N. IRAKLIOU | 21.4 (6.99) | 20.9 [4.21, 40.2] | 71.2 (12.4) | 73.0 [20.3, 95.8] |
| N. KARDITSAS | 19.6 (9.15) | 19.0 [-4.14, 41.6] | 67.8 (15.4) | 69.3 [26.1, 97.9] |
| N. KASTORIAS | 15.9 (9.40) | 15.8 [-12.4, 37.5] | 70.1 (14.9) | 71.0 [23.4, 99.5] |
| N. KAVALAS | 17.3 (7.60) | 16.7 [-3.23, 34.0] | 73.2 (10.5) | 74.0 [34.1, 96.1] |
| N. KEFALLONIAS | 18.2 (5.57) | 17.5 [0.983, 29.8] | 74.1 (10.0) | 75.9 [35.1, 93.9] |
| N. KERKYRAS | 18.4 (5.89) | 17.6 [0.603, 32.7] | 73.8 (9.92) | 75.6 [28.6, 95.8] |
| N. KILKIS | 19.3 (9.28) | 18.9 [-7.50, 42.2] | 67.3 (14.4) | 67.3 [27.9, 97.8] |
| N. KORINTHOU | 19.1 (7.95) | 18.4 [-2.49, 38.4] | 67.4 (12.5) | 68.4 [28.0, 96.1] |
| N. KOZANIS | 17.0 (9.34) | 16.9 [-10.4, 38.7] | 68.0 (15.3) | 68.1 [23.7, 99.3] |
| N. KYKLADON | 18.9 (5.24) | 18.3 [4.33, 29.7] | 72.8 (8.06) | 73.6 [42.3, 94.0] |
| N. LAKONIAS | 20.3 (7.62) | 19.3 [0.696, 40.0] | 68.1 (12.2) | 69.3 [22.8, 94.6] |
| N. LARISAS | 20.3 (9.06) | 19.8 [-2.54, 43.2] | 65.9 (15.2) | 66.8 [19.0, 98.0] |
| N. LASITHIOU | 20.0 (6.25) | 19.4 [3.80, 35.2] | 68.5 (8.23) | 68.8 [30.0, 90.8] |
| N. LEFKADAS | 18.7 (5.97) | 18.0 [1.07, 34.0] | 75.8 (9.88) | 78.0 [33.5, 96.1] |
| N. LESVOU | 18.9 (7.33) | 18.0 [-0.527, 34.5] | 70.3 (9.78) | 70.3 [40.7, 95.0] |
| N. MAGNISIAS | 18.0 (7.78) | 17.8 [-2.94, 40.5] | 71.2 (12.0) | 71.6 [25.9, 97.9] |
| N. MESSINIAS | 19.8 (7.54) | 18.7 [0.741, 39.8] | 72.2 (10.6) | 73.2 [28.0, 95.9] |
| N. PELLAS | 18.5 (9.26) | 18.3 [-9.71, 40.9] | 69.8 (14.3) | 70.3 [27.7, 98.8] |
| N. PIERIAS | 18.2 (7.81) | 17.9 [-4.87, 37.4] | 72.2 (11.4) | 72.5 [31.8, 96.4] |
| N. PIREOS KE NISON | 21.6 (8.23) | 20.6 [0.305, 42.7] | 66.4 (12.8) | 67.4 [26.7, 95.5] |
| N. PREVEZAS | 19.3 (7.45) | 18.4 [-0.378, 38.6] | 76.5 (9.93) | 77.6 [31.0, 97.4] |
| N. RETHYMNOU | 20.0 (6.95) | 19.4 [2.62, 38.3] | 69.6 (13.0) | 71.0 [19.2, 95.4] |
| N. RODOPIS | 18.4 (9.05) | 17.8 [-7.93, 37.6] | 70.7 (13.6) | 71.4 [31.1, 98.4] |
| N. SAMOU | 18.8 (5.81) | 18.3 [1.15, 31.5] | 70.7 (9.59) | 71.1 [43.2, 93.6] |
| N. SERRON | 19.4 (9.12) | 19.1 [-4.27, 41.1] | 68.7 (13.6) | 68.8 [30.1, 97.7] |
| N. THESPROTIAS | 18.6 (7.29) | 17.8 [-1.56, 38.5] | 75.6 (9.96) | 76.8 [30.2, 96.2] |
| N. THESSALONIKIS | 18.7 (8.50) | 18.4 [-6.08, 39.2] | 71.3 (11.8) | 71.4 [32.7, 97.1] |
| N. TRIKALON | 19.7 (9.35) | 19.2 [-4.84, 42.5] | 68.5 (15.5) | 69.5 [28.9, 98.9] |
| N. VIOTIAS | 20.0 (8.59) | 19.2 [-3.06, 42.0] | 68.6 (14.6) | 70.5 [23.8, 97.8] |
| N. XANTHIS | 17.3 (8.21) | 16.8 [-6.60, 35.5] | 72.9 (12.0) | 74.2 [34.5, 97.3] |
| N. ZAKYNTHOU | 18.9 (5.41) | 18.2 [2.36, 31.1] | 74.4 (9.36) | 75.8 [37.0, 93.9] |
| **Overall** | **19.7 (8.32)** | **19.1 [-14.0, 43.7]** | **69.4 (13.3)** | **70.7 [19.0, 99.8]** |

## Supplementary Table 2. Relative risk estimates for overall effect and effect modification by age and sex

|  |  | **HD1** | | **HD2** | | **HD3** | | **HD4** | | **HD5** | | **HD6** | |
| --- | --- | --- | --- | --- | --- | --- | --- | --- | --- | --- | --- | --- | --- |
| **Sex** | **Age group** | **Unadjusted RR (95%CrI)** | **Adjusted RR (95%CrI)** | **Unadjusted RR (95%CrI)** | **Adjusted RR (95%CrI)** | **Unadjusted RR (95%CrI)** | **Adjusted RR (95%CrI)** | **Unadjusted RR (95%CrI)** | **Adjusted RR (95%CrI)** | **Unadjusted RR (95%CrI)** | **Adjusted RR (95%CrI)** | **Unadjusted RR (95%CrI)** | **Adjusted RR (95%CrI)** |
| Total | Total | 1.12 (1.11, 1.12) | 1.08 (1.07, 1.09) | 1.11 (1.1, 1.12) | 1.07 (1.06, 1.09) | 1.11 (1.11, 1.12) | 1.08 (1.07, 1.1) | 1.13 (1.12, 1.14) | 1.09 (1.07, 1.1) | 1.2 (1.18, 1.21) | 1.13 (1.1, 1.15) | 1.23 (1.21, 1.26) | 1.15 (1.12, 1.19) |
|  | 65-74 | 1.05 (1.04, 1.06) | 1.04 (1.02, 1.06) | 1.05 (1.03, 1.06) | 1.03 (1.02, 1.05) | 1.05 (1.04, 1.07) | 1.04 (1.02, 1.06) | 1.06 (1.05, 1.08) | 1.05 (1.03, 1.07) | 1.11 (1.08, 1.13) | 1.08 (1.05, 1.11) | 1.14 (1.1, 1.17) | 1.11 (1.06, 1.15) |
|  | 75-84 | 1.13 (1.11, 1.14) | 1.08 (1.06, 1.1) | 1.12 (1.11, 1.13) | 1.08 (1.06, 1.1) | 1.12 (1.11, 1.13) | 1.08 (1.07, 1.1) | 1.14 (1.12, 1.15) | 1.09 (1.07, 1.11) | 1.21 (1.18, 1.24) | 1.14 (1.11, 1.18) | 1.25 (1.22, 1.29) | 1.19 (1.14, 1.24) |
|  | >=85 | 1.16 (1.15, 1.18) | 1.12 (1.1, 1.13) | 1.15 (1.14, 1.17) | 1.11 (1.09, 1.12) | 1.16 (1.15, 1.18) | 1.12 (1.1, 1.14) | 1.17 (1.16, 1.19) | 1.12 (1.09, 1.14) | 1.26 (1.23, 1.29) | 1.16 (1.12, 1.2) | 1.3 (1.26, 1.34) | 1.19 (1.13, 1.24) |
| Males | Total | 1.08 (1.07, 1.09) | 1.06 (1.04, 1.07) | 1.08 (1.07, 1.09) | 1.05 (1.04, 1.07) | 1.08 (1.07, 1.09) | 1.06 (1.04, 1.07) | 1.09 (1.08, 1.1) | 1.06 (1.05, 1.08) | 1.15 (1.13, 1.17) | 1.11 (1.08, 1.14) | 1.18 (1.15, 1.21) | 1.14 (1.1, 1.18) |
|  | 65-74 | 1.04 (1.03, 1.06) | 1.03 (1.01, 1.06) | 1.05 (1.03, 1.06) | 1.03 (1.02, 1.05) | 1.05 (1.03, 1.06) | 1.04 (1.02, 1.06) | 1.06 (1.04, 1.08) | 1.05 (1.03, 1.08) | 1.1 (1.06, 1.13) | 1.08 (1.04, 1.12) | 1.13 (1.08, 1.18) | 1.11 (1.05, 1.16) |
|  | 75-84 | 1.1 (1.08, 1.11) | 1.06 (1.04, 1.09) | 1.09 (1.07, 1.11) | 1.06 (1.03, 1.09) | 1.08 (1.06, 1.1) | 1.05 (1.03, 1.08) | 1.09 (1.07, 1.11) | 1.06 (1.04, 1.09) | 1.15 (1.12, 1.19) | 1.12 (1.08, 1.17) | 1.2 (1.15, 1.25) | 1.15 (1.1, 1.21) |
|  | >=85 | 1.13 (1.11, 1.15) | 1.08 (1.06, 1.11) | 1.11 (1.09, 1.14) | 1.08 (1.05, 1.1) | 1.12 (1.1, 1.14) | 1.08 (1.06, 1.11) | 1.12 (1.1, 1.15) | 1.08 (1.05, 1.11) | 1.21 (1.17, 1.25) | 1.14 (1.09, 1.19) | 1.24 (1.18, 1.3) | 1.17 (1.1, 1.24) |
| Females | Total | 1.15 (1.14, 1.16) | 1.11 (1.09, 1.12) | 1.14 (1.13, 1.15) | 1.1 (1.08, 1.11) | 1.15 (1.14, 1.16) | 1.11 (1.1, 1.13) | 1.17 (1.15, 1.18) | 1.12 (1.1, 1.14) | 1.25 (1.22, 1.27) | 1.15 (1.12, 1.19) | 1.29 (1.26, 1.32) | 1.19 (1.14, 1.23) |
|  | 65-74 | 1.07 (1.05, 1.09) | 1.04 (1.01, 1.07) | 1.05 (1.03, 1.07) | 1.03 (0.99, 1.06) | 1.07 (1.04, 1.09) | 1.05 (1.02, 1.08) | 1.06 (1.04, 1.09) | 1.04 (1.01, 1.08) | 1.12 (1.08, 1.17) | 1.08 (1.02, 1.13) | 1.15 (1.09, 1.22) | 1.1 (1.02, 1.18) |
|  | 75-84 | 1.16 (1.14, 1.17) | 1.11 (1.08, 1.13) | 1.16 (1.14, 1.17) | 1.11 (1.08, 1.13) | 1.16 (1.14, 1.18) | 1.12 (1.09, 1.14) | 1.18 (1.16, 1.2) | 1.13 (1.1, 1.15) | 1.27 (1.23, 1.31) | 1.19 (1.14, 1.23) | 1.31 (1.25, 1.36) | 1.21 (1.15, 1.29) |
|  | >=85 | 1.19 (1.17, 1.2) | 1.14 (1.12, 1.16) | 1.18 (1.16, 1.2) | 1.13 (1.11, 1.15) | 1.19 (1.17, 1.21) | 1.14 (1.12, 1.17) | 1.21 (1.19, 1.23) | 1.15 (1.12, 1.17) | 1.29 (1.26, 1.33) | 1.19 (1.14, 1.23) | 1.34 (1.29, 1.39) | 1.21 (1.14, 1.27) |

# Supplementary Figures

## Supplementary Figure 1. Annual heatwave intensity and frequency under each definition.

## Supplementary Figure 2. Total number of heatwave days under each definition over the study period in each NUTS3 region.

## Supplementary Figure 3. Median and 95% credible intervals of the posterior distribution of the relative heatwave risk from the fully adjusted model across the 6 heatwave definitions, using year- and region-specific thresholds.

HD1 at least 2 consecutive days with maximum temperature above the 90th percentile, HD2 at least 3 consecutive days above the 90th percentile, HD3 least 2 consecutive days with maximum temperature above the 95th percentile, HD4 at least 3 consecutive days above the 95th percentile, HD5 least 2 consecutive days with maximum temperature above the 99th percentile and HD6 at least 3 consecutive days above the 99th percentile.

## Supplementary Figure 4. Spatiotemporal variation of the heatwave effects across the 6 heatwave definitions using year- and region-specific thresholds. Panel A shows the median percentage change in the heatwave effect across the years, whereas panel B the posterior probability that the area-specific heatwave effect is larger than the overall nationwide effect across the different heatwave definitions. Both effects were based on the fully adjusted models.

HD1 at least 2 consecutive days with maximum temperature above the 90th percentile, HD2 at least 3 consecutive days above the 90th percentile, HD3 least 2 consecutive days with maximum temperature above the 95th percentile, HD4 at least 3 consecutive days above the 95th percentile, HD5 least 2 consecutive days with maximum temperature above the 99th percentile and HD6 at least 3 consecutive days above the 99th percentile.

## Supplementary Figure 5. Numbers of heatwave days (y-axis) in factual and counterfactual scenarios over the study period across (x-axis) the 6 heatwave definitions using year- and region-specific thresholds.

HD1 at least 2 consecutive days with maximum temperature above the 90th percentile, HD2 at least 3 consecutive days above the 90th percentile, HD3 least 2 consecutive days with maximum temperature above the 95th percentile, HD4 at least 3 consecutive days above the 95th percentile, HD5 least 2 consecutive days with maximum temperature above the 99th percentile and HD6 at least 3 consecutive days above the 99th percentile.

## Supplementary Figure 6. Median and 95% credible intervals of the number of heatwave-deaths and relative difference of deaths due to human-induced climate change across the study period, with heatwave definitions using year- and region-specific thresholds.

HD1 at least 2 consecutive days with maximum temperature above the 90th percentile, HD2 at least 3 consecutive days above the 90th percentile, HD3 least 2 consecutive days with maximum temperature above the 95th percentile, HD4 at least 3 consecutive days above the 95th percentile, HD5 least 2 consecutive days with maximum temperature above the 99th percentile and HD6 at least 3 consecutive days above the 99th percentile.

## Supplementary Figure 7. Heatwave effects across lag0 to lag3 and the common-effect lag0–3 estimate for six heatwave definitions

## Supplementary Figure 8. Comparison of common-effect and jointly modelled cumulative lag specifications

## Supplementary Figure 9. Temporal heatwave effects under alternative prior specifications: first-order random walk and zero-mean normal prior with unstructured covariance

# Supplementary Texts

## Supplementary Methods Section 1: Justification of population-weighted temperature

directly averaging the temperature across NUTS3 regions does not adequately represent the actual exposure experienced by the populations. This limitation arises from two aspects. First, temperature is retrieved at gridded level, while the mortality data are aggregated at NUTS3 region level, which causes spatial misalignment. Moreover, populations are not evenly distributed across space. Urban areas are usually densely populated, while some areas such as forests or mountains may be sparsely populated or uninhabited. For each NUTS3 region, we calculated its maximum daily temperature as a population-weighted average of the grid cell temperatures, using the same year’s gridded population data from WorldPop at 1 km geographical resolution ([www.worldpop.org](http://www.worldpop.org/)).

$$T_{weighted}=\frac{\sum T_{grid}P_{grid}}{\sum P_{grid}}$$

## Supplementary Methods Section 2: Counterfactual temperatures

The change in heatwave temperatures in each region associated with each degree of global warming was estimated using the probabilistic attribution protocol established by World Weather Attribution (1,2). We characterise heatwave temperatures in each region using Tx3x, the average of the daily maximum temperatures during the hottest three-day period in each year. A nonstationary Generalised Extreme Value (GEV) distribution is fitted to this time series of annual maxima, in which the location parameter of the distribution is assumed to shift linearly with global mean surface temperature (GMST), while the scale and shape parameters remain constant, so that

$Tx3x GEV\left( \mu,\sigma| \mu_{0},\sigma_{0},\xi,\alpha,T \right)$ with $\mu=\mu_{0}+\alpha T$ and $\sigma=\sigma_{0}$,

Where $\mu,\sigma,\xi$ are the location, scale and shape parameters, *T* is the GMST, and $\alpha$ is the change in local temperature per degree of GMST increase. To remove interannual variability, the GMST covariate is first smoothed using a 4-year running window. The parameters of this statistical model are obtained via maximum likelihood estimation. 95% confidence intervals are obtained by bootstrapping, using a sample size of 1000.

This procedure is repeated for two different observation-based datasets - E-Obs, which uses interpolated station data (3), and ERA5-land, a forecast-based reanalysis product(4) - and for 72 regional climate model runs from the Euro-CORDEX ensemble (5,6). The estimated $\alpha$ parameters from all of these data sources are then combined into a single, overarching estimate of the attributable change in local temperatures per degree of global warming, along with synthesised 95% confidence intervals, following the procedure described previously (7). This is done by first computing the weighted average of the estimates from the two observational datasets and, independently, the weighted average of the estimates from the 72 climate models; and then by calculating the weighted average of the two.

Having estimated the change in local heatwave temperatures per degree of global warming in this way, we calculate how much warmer each year was than a preindustrial climate with no or minimal human-caused warming (assumed to be 1.3°C cooler than 2025’s mean temperature, per the Global Warming Index(8)). This temperature offset is multiplied by the alpha parameter and the estimated upper and lower bounds for each region to obtain an estimate and interval for local warming attributable to climate change. Finally, we subtract this attributable warming from the daily maximum temperatures in the ERA5-land dataset to obtain a counterfactual time series, along with upper and lower bounds. These three counterfactual time series are used as inputs to the epidemiological model, along with the factual time series (daily maximum temperatures averaged over each region from ERA5-land).

## Supplementary Methods Section 3: Death attribution

Omitting subscripts for simplicity, let $\tilde{D}$ be the mean number of deaths during a heatwave day, *M* the number of heatwave days and RR the relative heatwave risk, then:

$$AN=\frac{RR-1}{RR}*(M*\tilde{D})$$

Let $AN_{f}$ denote the number of deaths in the *factual* scenario (based on the *observed* number of heatwave days $M_{f}$), and $AN_{c}$ the number of deaths in the counterfactual scenario without human-induced climate change (where the number of heatwave days $M_{c}$ is calculated using the counterfactual temperature time series). We report the median and 95% CrI for both the number and proportion of heatwave-related deaths attributable to climate change, defined respectively as $AN_{f}-AN_{c}$, and $(AN_{f}-AN_{c})/AN_{f}$. This proportion should be interpreted as the share of the factual heatwave-attributable mortality burden that would not have occurred under the counterfactual climate, rather than as an epidemiologic attributable fraction reflecting the magnitude of the heatwave relative risk.

The heatwave-related relative mortality risks were estimated using thresholds derived from the factual temperature series to ensure consistency in the relative risks.

**Supplementary Reference**

1.	Van Oldenborgh GJ, Van Der Wiel K, Kew S *et al.* Pathways and pitfalls in extreme event attribution. *Climatic Change* 2021;**166**(1–2):13. https://doi.org/10.1007/s10584-021-03071-7.

2.	Philip S, Kew S, Van Oldenborgh GJ *et al.* A protocol for probabilistic extreme event attribution analyses. *Adv Stat Clim Meteorol Oceanogr* 2020;**6**(2):177–203. https://doi.org/10.5194/ascmo-6-177-2020.

3.	Cornes RC, Van Der Schrier G, Van Den Besselaar EJM *et al.* An Ensemble Version of the E‐OBS Temperature and Precipitation Data Sets. *JGR Atmospheres* 2018;**123**(17):9391–409. https://doi.org/10.1029/2017JD028200.

4.	Hersbach H, Bell B, Berrisford P *et al.* The ERA5 global reanalysis. *Quart J Royal Meteoro Soc* 2020;**146**(730):1999–2049. https://doi.org/10.1002/qj.3803.

5.	Jacob D, Petersen J, Eggert B *et al.* EURO-CORDEX: new high-resolution climate change projections for European impact research. *Reg Environ Change* 2014;**14**(2):563–78. https://doi.org/10.1007/s10113-013-0499-2.

6.	Vautard R, Kadygrov N, Iles C *et al.* Evaluation of the Large EURO‐CORDEX Regional Climate Model Ensemble. *JGR Atmospheres* 2021;**126**(17):e2019JD032344. https://doi.org/10.1029/2019JD032344.

7.	Otto FEL, Barnes C, Philip S *et al.* Formally combining different lines of evidence in extreme-event attribution. *Adv Stat Clim Meteorol Oceanogr* 2024;**10**(2):159–71. https://doi.org/10.5194/ascmo-10-159-2024.

8.	globalwarmingindex.org -- Tracking progress to a safe climate. n.d. https://www.globalwarmingindex.org/ (5 Dec. 2025, date last accessed).
